# Supplementary material for: Multiplexed profiling of transcriptional regulators in plant cells
Source: Nat Biotechnol. 2025 Nov 25;44(11):1920–33. doi: 10.1038/s41587-025-02880-w (PMC13751309; doi:10.1038/s41587-025-02880-w)
Supplement: Supplementary file 1 [file 41587_2025_2880_MOESM1_ESM.pdf]

nature biotechnology

Supplementary information

https://doi.org/10.1038/s41587-025-02880-w

Multiplexed profiling of transcriptional regulators in plant cells

In the format provided by the authors and unedited

Table of contents

1. **Supplementary text: Resolution and reproducibility of ENTRAP-seq measurements.**
2. **Supplementary Figures 1-11**
3. **Supplementary table 1: plasmids**
4. **Supplementary table 2: primers**

Resolution and reproducibility of ENTRAP-seq measurements

September 19, 2025

1 Introduction

The goal of this section is to understand the resolution of ENTRAP-seq measurements in terms of the transcriptional activity or 'strength' of different proteins. Given two proteins that have different sequencing enrichment values we want to answer the question of how confident are we that their strengths are actually quantitatively different. Intuitively, our confidence will depend on how large is the difference between their enrichment values, Δ. For example, a protein tile with an enrichment of 0.5 is almost certainly a weaker activator than a tile with an enrichment of 1.5. The situation is trickier when comparing measurements with quantitatively subtle differences in activity ($\Delta < b$, for some constant b). To approach this problem we need to incorporate the measurement error into the analysis in order to arrive at a more definite answer of the kind *'The difference in enrichment of two proteins needs to be $\Delta \geq b$ for us to say that they are different with a confidence of $p \leq 0.05$'*. Here we followed a nonparametric approach to model the error in order to reach this kind of claim about quantitative uncertainty. Finally, we use these analyses to understand the quantitative resolution of the data presented in the manuscript. We find that for a single replicate of the largest library (2,000 tiles) the resolution is approximately $\pm$ 0.45 enrichment units. When two replicates are combined to calculate the mean enrichment per tile, the resolution improves to approximately $\pm$ 0.3 enrichment units.

2 Non-parametric approach to estimate measurement uncertainty

We seek to answer three questions:

1. What is the minimum difference in enrichment between two proteins that one can confidently detect in a single replicate?
2. What is the uncertainty or confidence interval around the enrichment of a single protein when doing two replicates?
3. What is the minimum difference in enrichment between two proteins that one can confidently detect when using two replicates?

Our approach to answer these questions will be based on calculating a "null distribution", a statistical concept that defines what values of a measurement (e.g., a difference) one can expect under the assumption that there is no real effect — that is, under the null hypothesis that there is no difference. The logic is that values on the tail ends of this distribution are unlikely to be random noise.

2.1 Resolution for the difference in activity between two proteins in a single replicate

If each library is measured more than once, it becomes possible to construct an empirical null distribution of enrichment differences between two measurements of the *same* protein. These differences are, by definition, attributable to measurement noise, since the underlying biological signal (the protein's transcriptional strength) is constant.

This empirical distribution captures the magnitude of variability we should expect when comparing two proteins that are in fact, equal in transcriptional strength. Therefore, if the observed difference in enrichment between two different proteins greatly exceeds this noise distribution, we can infer that such a difference is unlikely to have arisen by chance — and is more likely to reflect a true underlying difference in transcriptional activity.

To obtain this empirical experimental noise distribution we calculate Δ, the delta in activity of the ith protein in replicate 1 and 2 as

$$\Delta_i = x_{i,1} - x_{i,2}. \tag{1}$$

With the values of Δx for all proteins we can then build a distribution D which is symmetrical, centered around 0 and with an empirical standard deviation of σ_Δ (Fig. S7A).

When the true biological activity of two proteins is identical, we expect their difference in enrichment to fall within this distribution D. More specifically, 95% of the time the Δx of two proteins identical in activity will fall within $0 \pm 2\sigma_{\Delta x}$. It follows that if the absolute difference in enrichment between two proteins exceeds $2\sigma_{\Delta x}$ these proteins have a probability $p \leq 0.05$ to be equal in activity.

We call $2\sigma_{\Delta x}$ the minimal detectable difference in activity between two proteins in a single replicate of an ENTRAP-seq experiment. Hence, when dealing with at a single replicate, we can claim with 95% confidence that protein A and protein B with enrichment values x_a and x_b are different if

$$|x_a - x_b| \geq 2\sigma_\Delta. \tag{2}$$

Without applying any coverage cutoffs or data normalizations, $2\sigma_{\Delta x} \approx 0.45$ (Fig. S7A).

Since we defined a threshold for activation at an enrichment = 1 and the maximum enrichment values are ≈ 2.5, we can define

$$\text{dynamic range of activation} \approx 2.5 - 1 \approx 1.5 \text{ enrichment units.} \tag{3}$$

Dividing this dynamic range by the assay resolution gives us an idea of how many quantitatively distinct groups of activators we can reliably distinguish.

$$\text{distinguishable groups} \approx \frac{\text{dynamic range in enrichment}}{\text{resolution}} \approx \frac{1.5}{2\sigma_\Delta} \approx 3 \tag{4}$$

Thus, in a single replicate ENTRAP-seq can detect at most 4 groups: not activators (enrichment ≤ 1) and weak, medium, and strong activators, with enrichment ranges 1-1.5, 1.5-2, and $\geq$2, respectively. Note however, that this result assumes that we have only one replicate: we only used the two replicates to estimate the experimental error but did not take advantage of the extra information provided by averaging the activity across replicates. In the next section we look at the assay resolution when 2 or more replicates are considered.

2.2 Assay resolution incorporating replicates

In principle, noise should scale as $1/\sqrt{n}$ for n replicates. Hence, we expect the resolution of ENTRAP-seq to improve from 0.45 to ~ 0.3 enrichment units by adding one replicate. The following derivation demonstrates how the uncertainty is reduced by adding a second replicate.

For each protein we have multiple measurements, x_{ij} for the ith protein in the jth replicate. Let's consider two proteins A and B and two replicates. Then the mean enrichment $\bar{x}$ of each protein is

$$\bar{x}_a = \frac{x_{a1} + x_{a2}}{2}, \ \bar{x}_b = \frac{x_{b1} + x_{b2}}{2}, \tag{5}$$

The difference in enrichment between these two proteins, Δx is therefore

$$\Delta x = \bar{x}_a - \bar{x}_b. \tag{6}$$

What we are after is the uncertainty or standard error (SE) in the difference between these two means,

$$\text{uncertainty} = SE_{\Delta}, \tag{7}$$

which can be obtained by propagating σ_x, the error associated with $\bar{x}_a$ and $\bar{x}_b$. Note that we are assuming that the error is the same across all proteins (known as homoscedasticity), an assumption that is well supported by our data (Fig. S7E,F). From the formula of error propagation of a sum, we have that the error in the subtraction of two measurements with an associated uncertainty is given by

$$SE_{\Delta x} = \sqrt{\frac{\sigma_x^2}{2} + \frac{\sigma_x^2}{2}} = \sqrt{\frac{\sigma_x^2}{1}} = \sigma_x. \tag{8}$$

Now we turn our attention to obtaining σ_x. Since σ_x is the standard error of x_i with respect to $\bar{x}_i$, we can estimate it by considering the residuals r_i of the data with respect to the y=x line defined by replicate 1 = replicate 2

$$r_i = \frac{1}{\sqrt{2}}(x_{i,1} - x_{i,2}), \tag{9}$$

where the $\sqrt{2}$ arises from the geometric projection of the residuals orthogonally onto x=y. Visually, these residuals can be obtained from plotting one replicate against the other and then taking the orthogonal distance of each observation to the y=x line. In the absence of any bias between replicates, r_i should be approximately normally distributed around a mean of 0 (Fig. S7C). This distribution of residuals with an empirical standard error of σ_ε now allows us to estimate σ_x. More specifically, we can be 95% certain that the value of σ_x lies within $2\sigma_\varepsilon$. Thus, for the purpose of estimating the uncertainty associated with comparing the mean enrichment between two proteins we have that

$$SE_\Delta = 2\sigma_\varepsilon, \tag{10}$$

meaning that we can be 95% sure that two proteins are biologically distinct in activity if

$$|\bar{x}_a - \bar{x}_b| \geq 2\sigma_\varepsilon. \tag{11}$$

In our datasets, without any sort of normalization or coverage cutoffs, $\sigma_\varepsilon \approx 0.15$, resulting in a minimal detectable difference in activity of approximately 0.3 enrichment units (Fig. S7C,D). This resolution is ~0.15 units better than using a single replicate, or 40% better. (see previous section: Resolution for the difference in activity between two proteins in a single replicate). Following the reasoning we described in the previous section, we have that

$$\text{distinguishable groups} \approx \frac{\text{dynamic range in enrichment}}{\text{resolution}} \approx \frac{1.5}{2\sigma_\varepsilon} \approx 5 \tag{12}$$

This number can be interpreted as ENTRAP-seq distinguishing 5-6 different classes of proteins: not activators (enrichment $\leq$1) and 5 different strength classes of activators of increasing potency.

3 Conclusions

Here, to estimate the uncertainty associated with replicate measurements, we derived an empirical error distribution (also known as the null distribution) from the observed differences between repeated measurements of the same protein (i.e., replicate enrichment values).

The standard deviation (σ) of this distribution was used directly—without assuming normality—to define detection thresholds and confidence intervals.

This non-parametric approach is conceptually aligned with the Bayesian emphasis on data-driven error estimation discussed in Data Analysis: A Bayesian Tutorial (Sivia & Skilling, 2006), where parameter-free inference is preferred when theoretical assumptions are weakly justified. Our use of the empirical 95th percentile as a confidence threshold also follows conventions in robust statistics and is compatible with techniques discussed in modern data analysis texts (e.g., Wasserman, 2006).

In contrast to parametric approaches that assume a Gaussian error model, our method reflects the actual noise structure of the dataset, capturing subtle deviations such as thinner-tailed distributions (as revealed by Anderson–Darling tests). This is particularly useful when the noise is symmetrical but not normally distributed.

This analysis revealed that ENTRAP-seq can reliably identify quantitative differences in enrichment among activators but like any method, the resolution is limited by experimental noise. With a single replicate the resolution is approximately 0.45 enrichment units, sufficient to distinguish 3 quantitative classes of activator strengths. This resolution is improved by adding a second replicate, in which case ENTRAP-seq can distinguish about 5 quantitative classes of activators.

In either case, the resolution depends directly on the noise and can therefore be improved by reducing the experimental error. As shown in the main text, a straightforward way to reduce noise is by imposing library coverage cutoffs. This makes intuitive sense, as library elements that are underrepresented in the library end up being represented in fewer cells, making their measurement subject to more cell-to-cell heterogeneity in transgene expression. Indeed, by applying a moderate coverage cutoff that retains the 75% most abundant tiles it is possible to improve the resolution of a single replicate from 0.45 to 0.3-0.4. For two replicates, a similar coverage cutoff improves the resolution to ~ 0.2 enrichment units, a fine enough resolution to distinguish multiple quantitative levels of TF strength. An interesting conclusion from this is that more homogeneous libraries should improve the assay resolution.

4 References

Sivia, D. S., & Skilling, J. (2006). Data Analysis: A Bayesian Tutorial (2nd ed.). Oxford University Press. ISBN: 9780198568322.

Wasserman, L. (2006). All of Nonparametric Statistics. Springer. https://doi.org/10.1007/0-387-30623-4

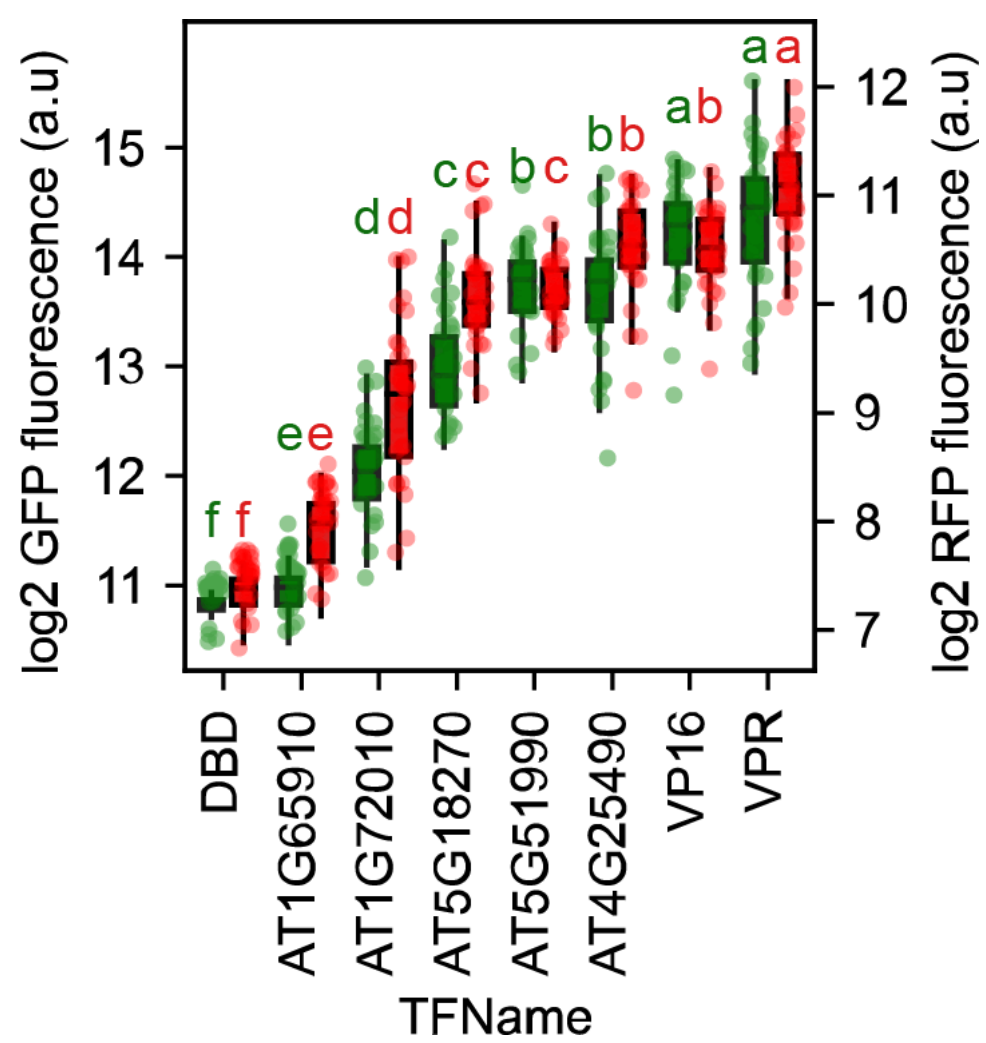

Figure S1: Related to Figure 1G. Sensitivity of RFP-T2A-SlWIP2-C as a fluorescence reporter. Shown is the same data as Fig. 1 G. Fluorescence of leaves infiltrated with the 6xUAS-min35S-RFP-T2A-SlWIP2-C reporter in combination with Gal4DBD fusions with different TFs or activation domains (same data as in Fig. 1G). Box plots show median ± IQR, whiskers correspond to minimum and maximum values, excluding outliers. The letters on top of each box represent statistically significant groups based on a post-hoc Tuckey Honestly Significant Difference (HSD) test performed separately for GFP and RFP. As in Fig. 1 G, N = 24 biological replicates (3 plants, 2 leaves per plant, 4 leaf punches per plant).

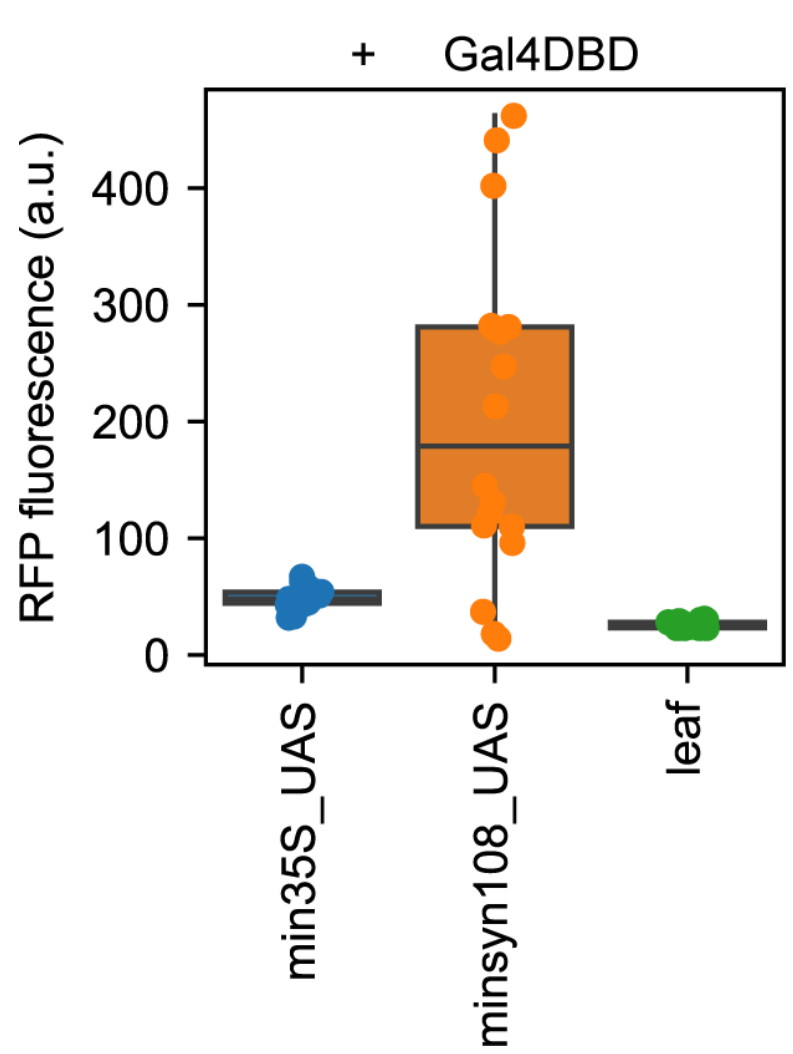

Fig. S2: Basal expression level of different minimal promoters. Box plot of tissue-level RFP fluorescence of leaves infiltrated with Gal4DBD and either one of two minimal promoters driving RFP-T2A-SlWIP2-C NTF. The min35S_UAS promoter corresponds to the minimal -46 35S promoter containing 6 upstream Gal4 binding sites. The minsyn108_UAS promoter is a modified version of the previously published minsyn108 promoter containing 6 embedded Gal4 binding sites without deleting original sequences. Leaf autofluorescence is shown as a reference. The min35S_UAS promoter was used for all ENTRAP-seq experiments and for fluorescence

reporter assays targeting activators. The minsyn108_UAS promoter was used for fluorescence assays to measure repressors in Fig.2F, Fig.3B. Boxes show median ± IQR, whiskers correspond to minimum and maximum values. N = 24 biological replicates (3 plants, 2 leaves per plant, 4 leaf punches per plant).

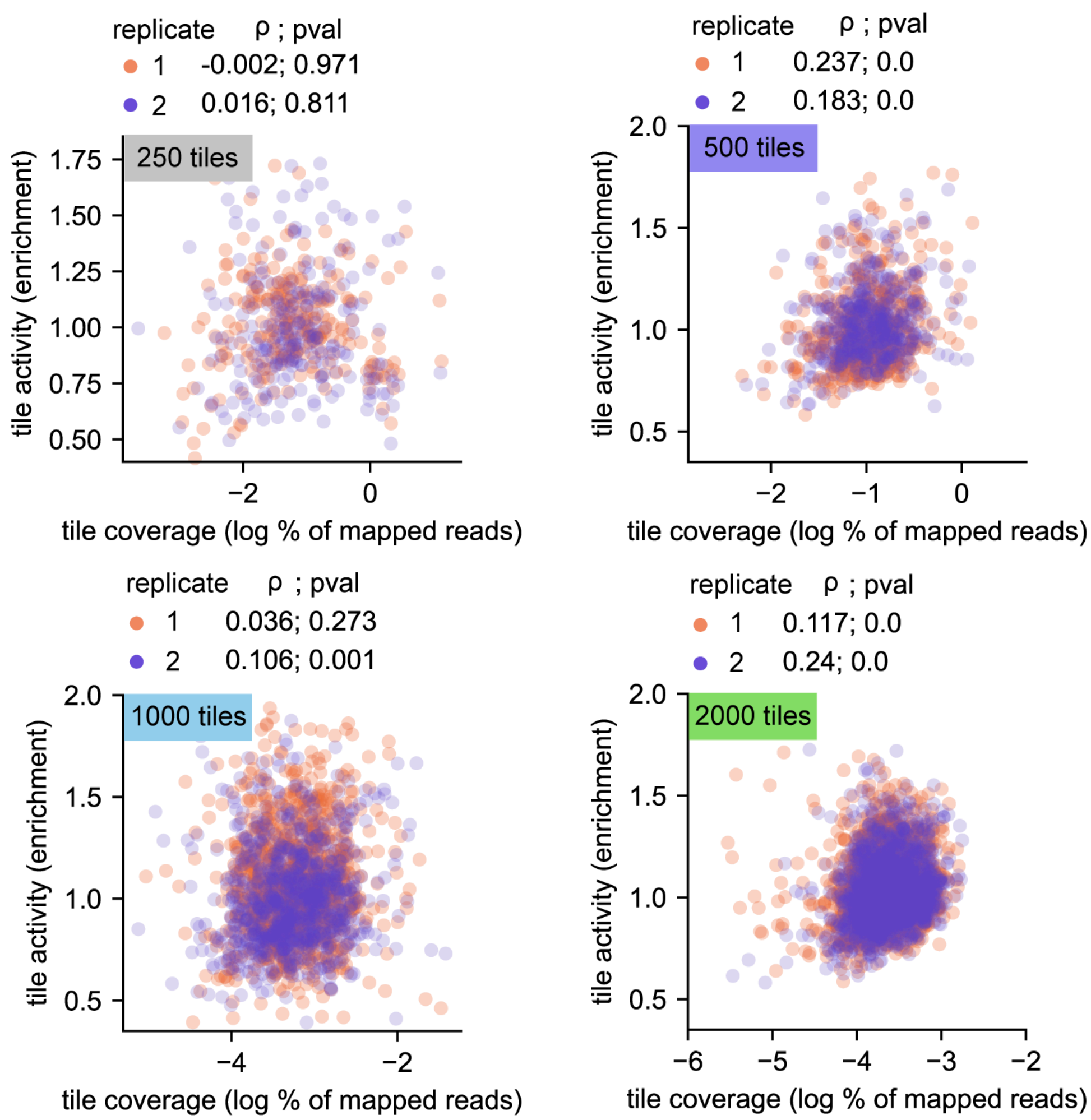

Figure S3: No correlation between tile coverage in the library and enrichment ratio. Scatter plots showing the transcriptional activity of each tile as measured by its enrichment value (y axis) as a function of the abundance of this tile in the library (x axis).

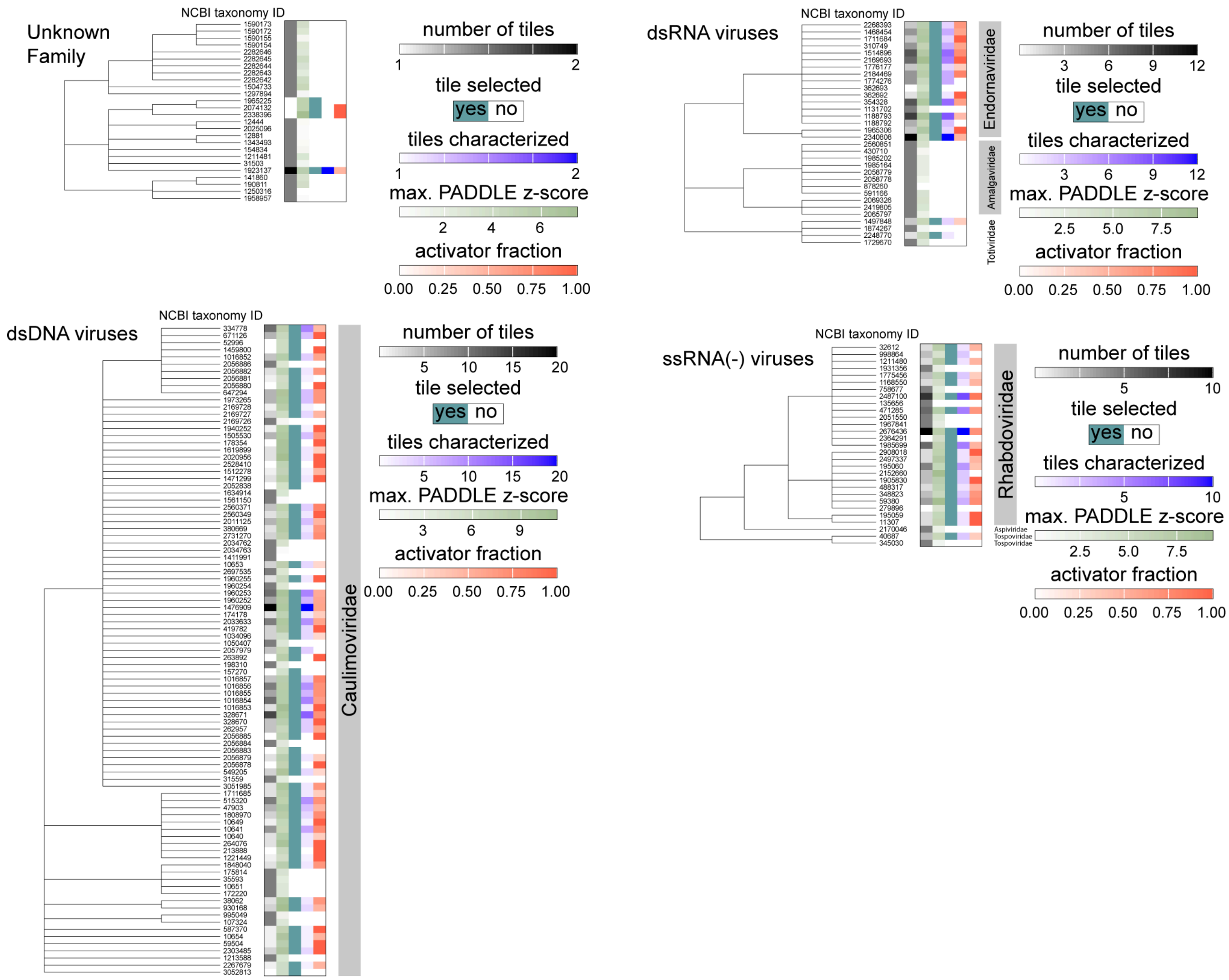

Figure S4: Details for double-stranded RNA, double-stranded DNA, single-tranded negative RNA, and unknown virus families. Shown are zoomed-in segments from the phylogenetic tree plot in Figure 4A. See also Table S1: Compiled data for viral libraries.

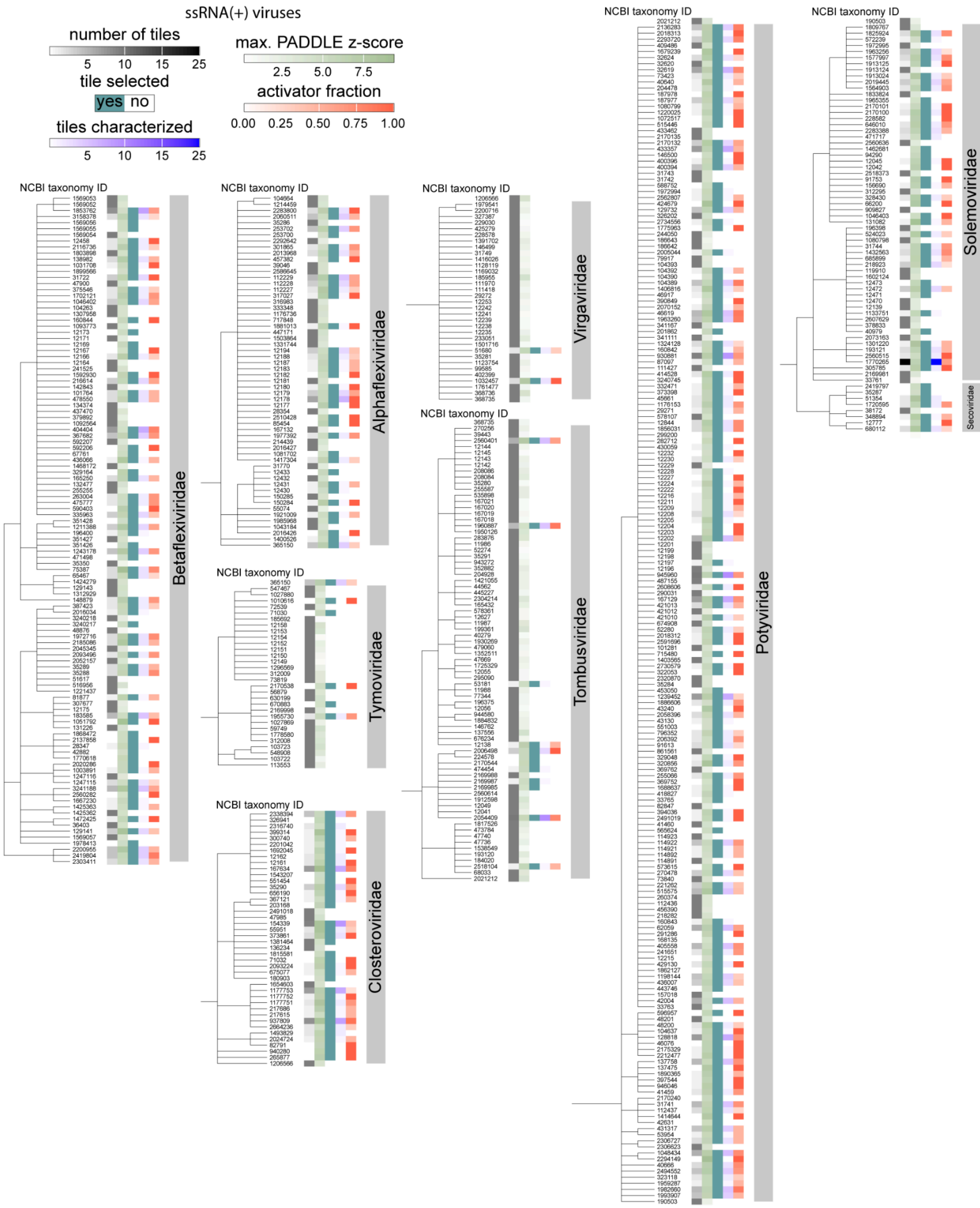

Figure S5: Details for positive single-stranded RNA virus families. Shown are zoomed-in segments from the phylogenetic tree plot in Figure 4A. See also Table S1: Compiled data for viral libraries.

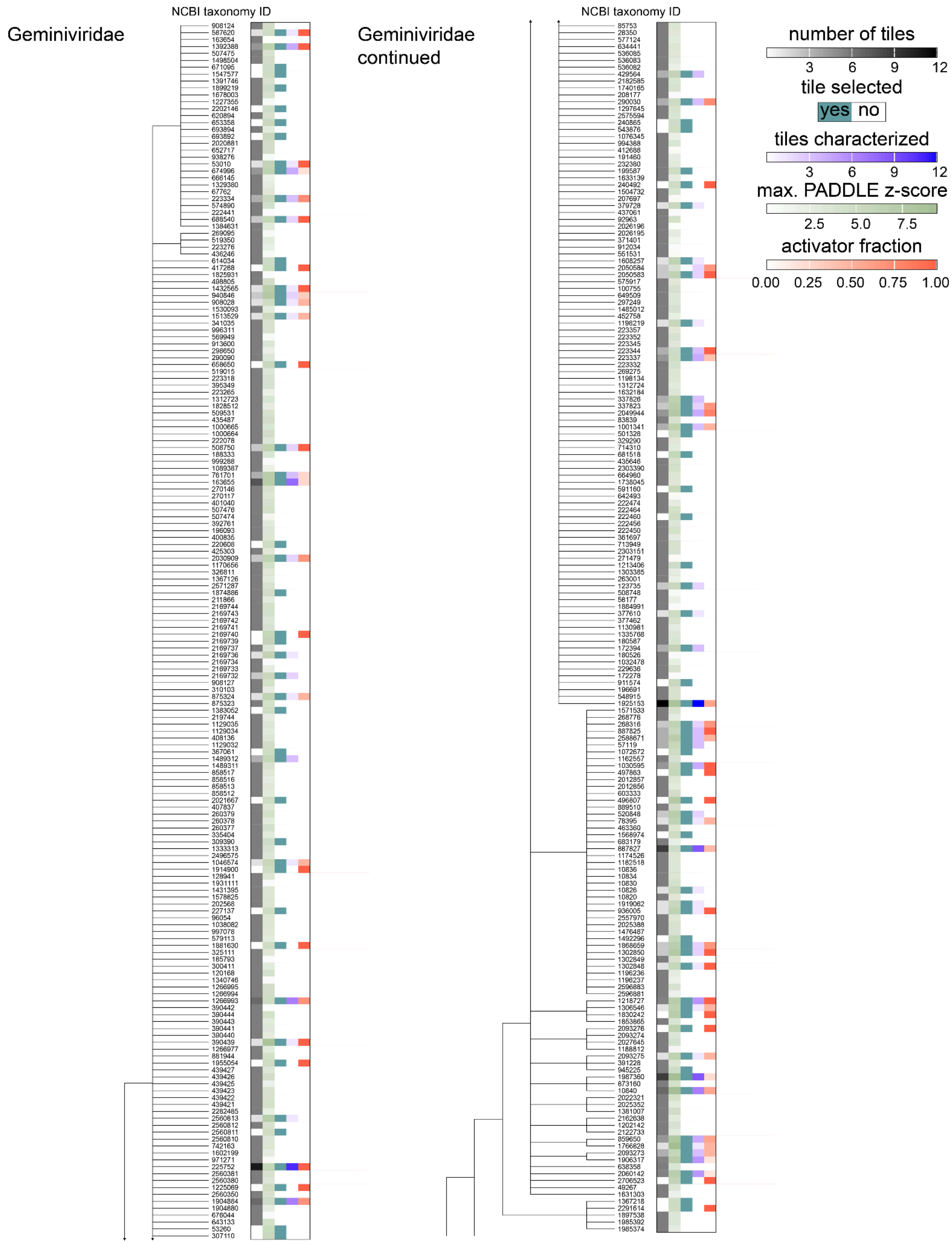

Figure S6: Details for *Geminiviridae*. Shown are zoomed-in segments from the phylogenetic tree plot in Figure 4A corresponding to viruses in the *Geminiviridae* family of single-stranded DNA viruses. See also Table S1: Compiled data for viral libraries.

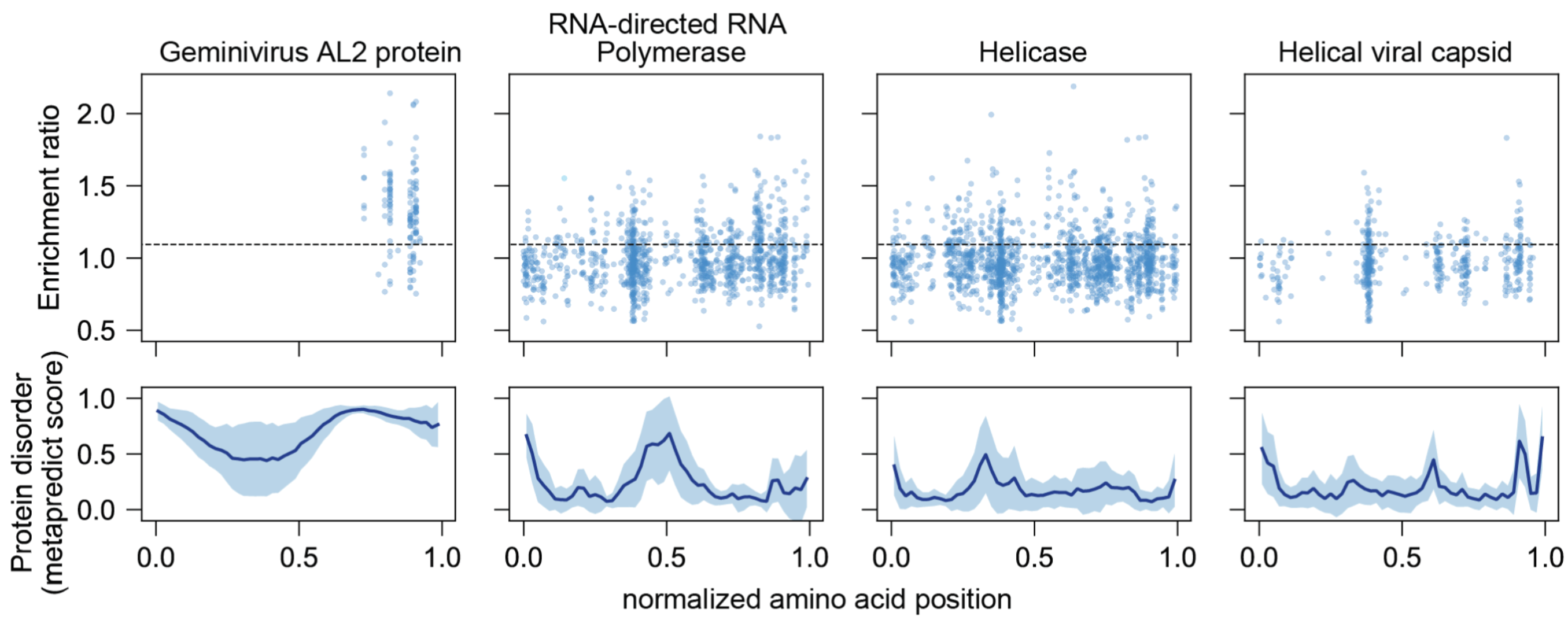

Figure S7: Enrichment ratios for tested tiles from four overrepresented protein classes. Top: Enrichment ratio of each tile along the (normalized) length of all proteins belonging to each given class. The amino acid position of each tile was normalized by dividing by the length of each corresponding protein. The horizontal dashed line shows the maximum threshold across datasets used to classify tiles as activators. Bottom: average ± 1 s.d. protein disorder as predicted by Metapredict.

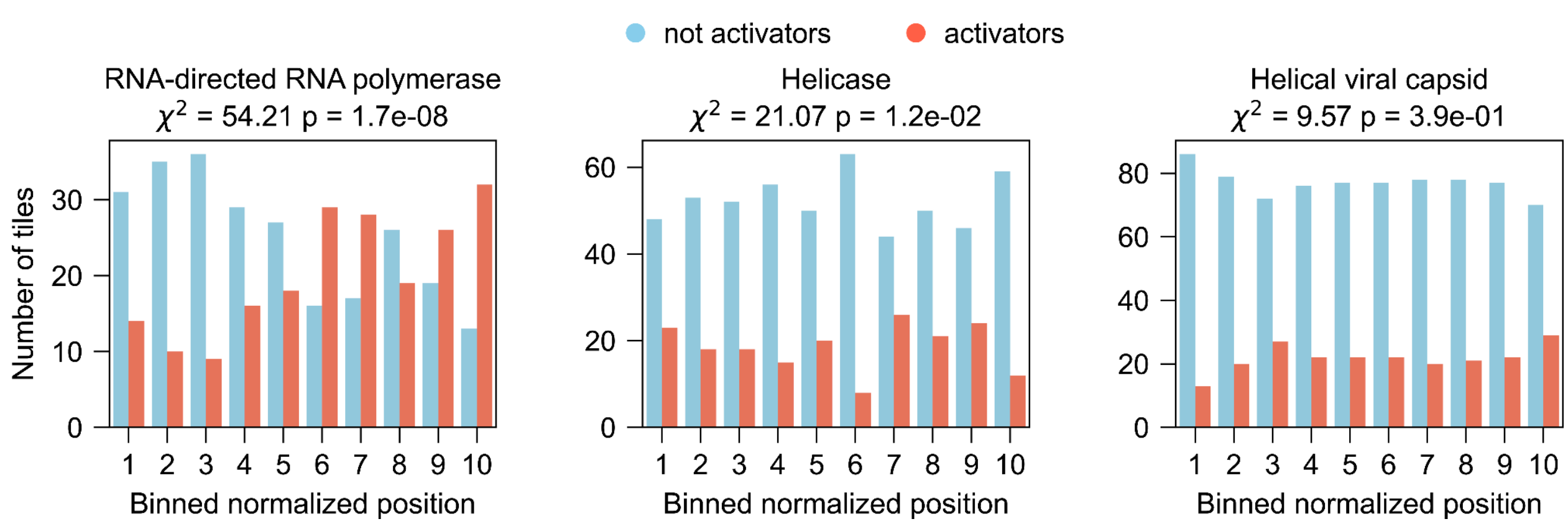

Figure S8: Positional clustering of putatively identified ADs. Normalized protein positions were binned and the distribution of identified AD regions showed statistically significant clustering in RdRp and helicase proteins, but not for helical capsid proteins, as determined by a chi-squared test.

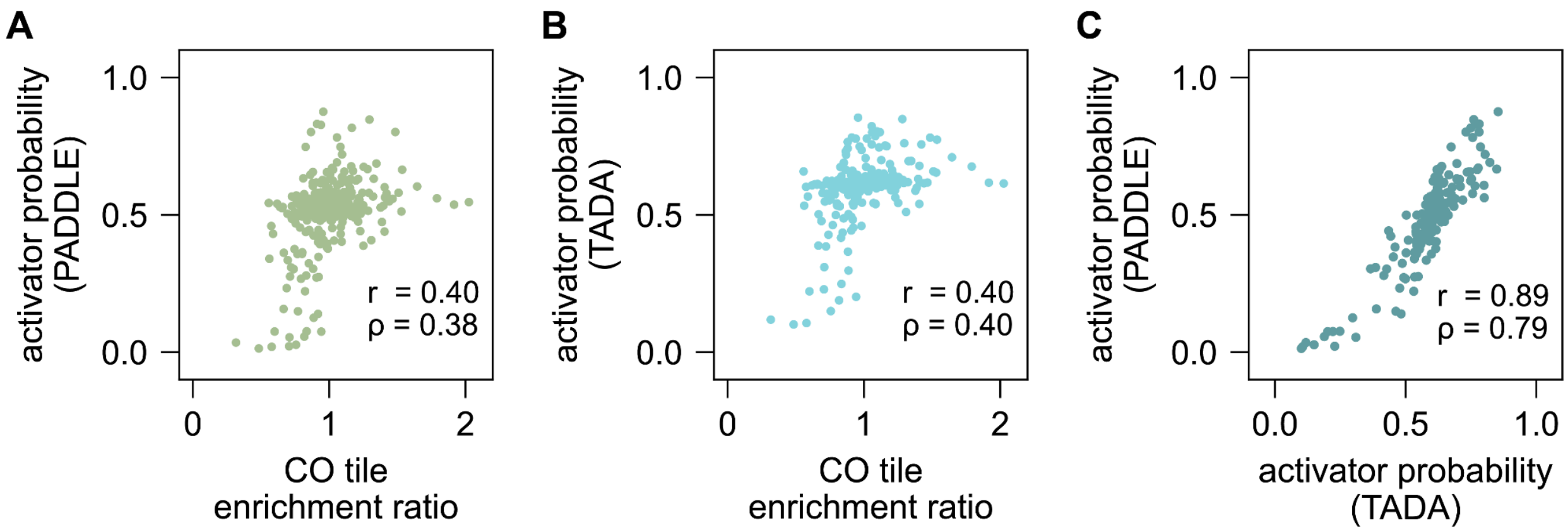

Figure S9: CO variant inference using PADDLE and TADA. (A) Median activator predictions from PADDLE and **(B)** TADA for each CO tile correlates with enrichment ratio. **(C)** Median PADDLE and TADA predictions for each CO tile also show strong concordance with respect to one another.

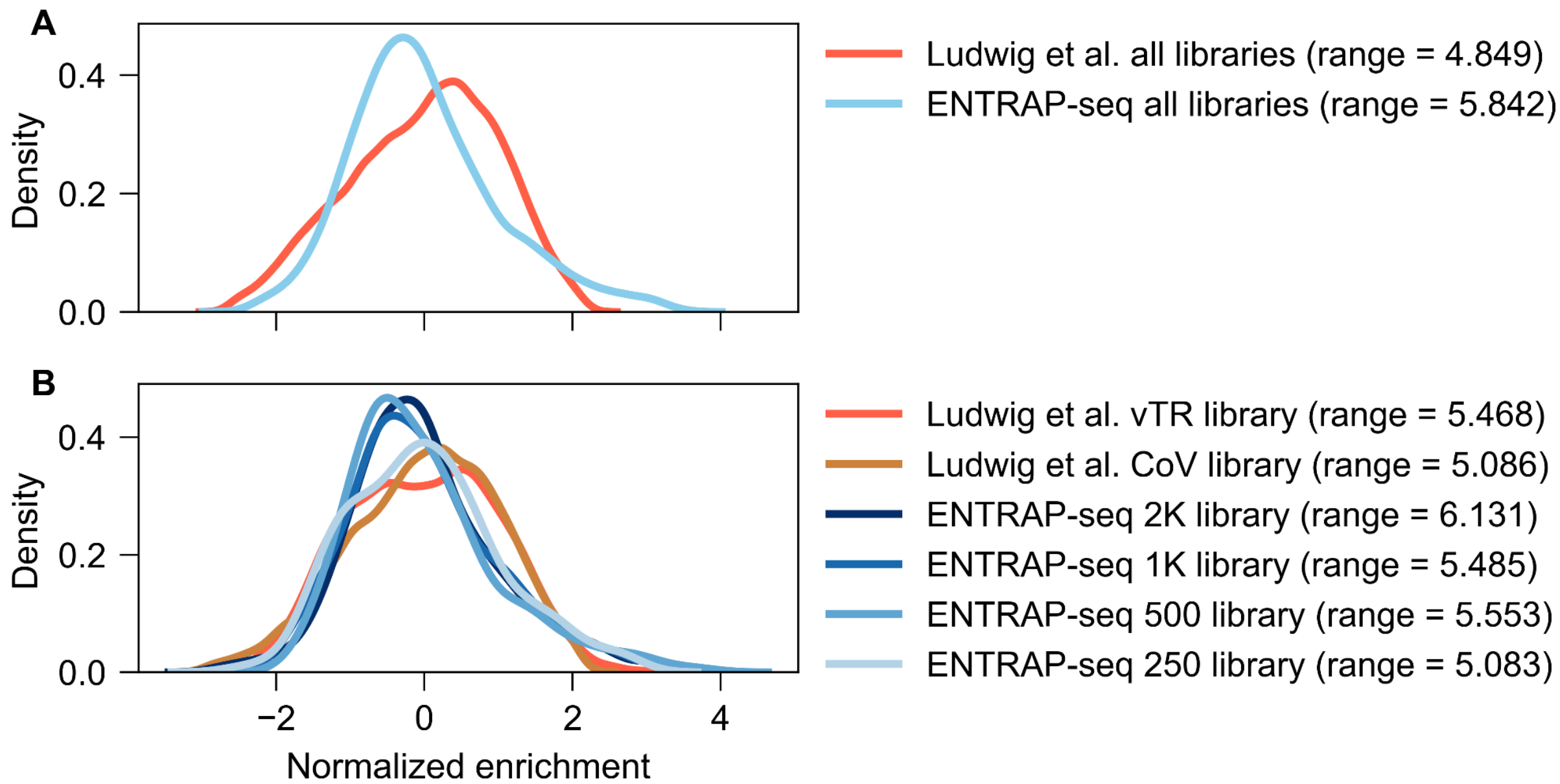

Figure S10: Dynamic range comparison between Ludwig et al. and ENTRAP-seq. Dynamic range was assessed after z-score normalization and excluding outliers above the 99th and below the 1st percentile in **(A)** all Ludwig et al. and ENTRAP-seq libraries as well as **(B)** individual Ludwig et al. and ENTRAP-seq libraries.

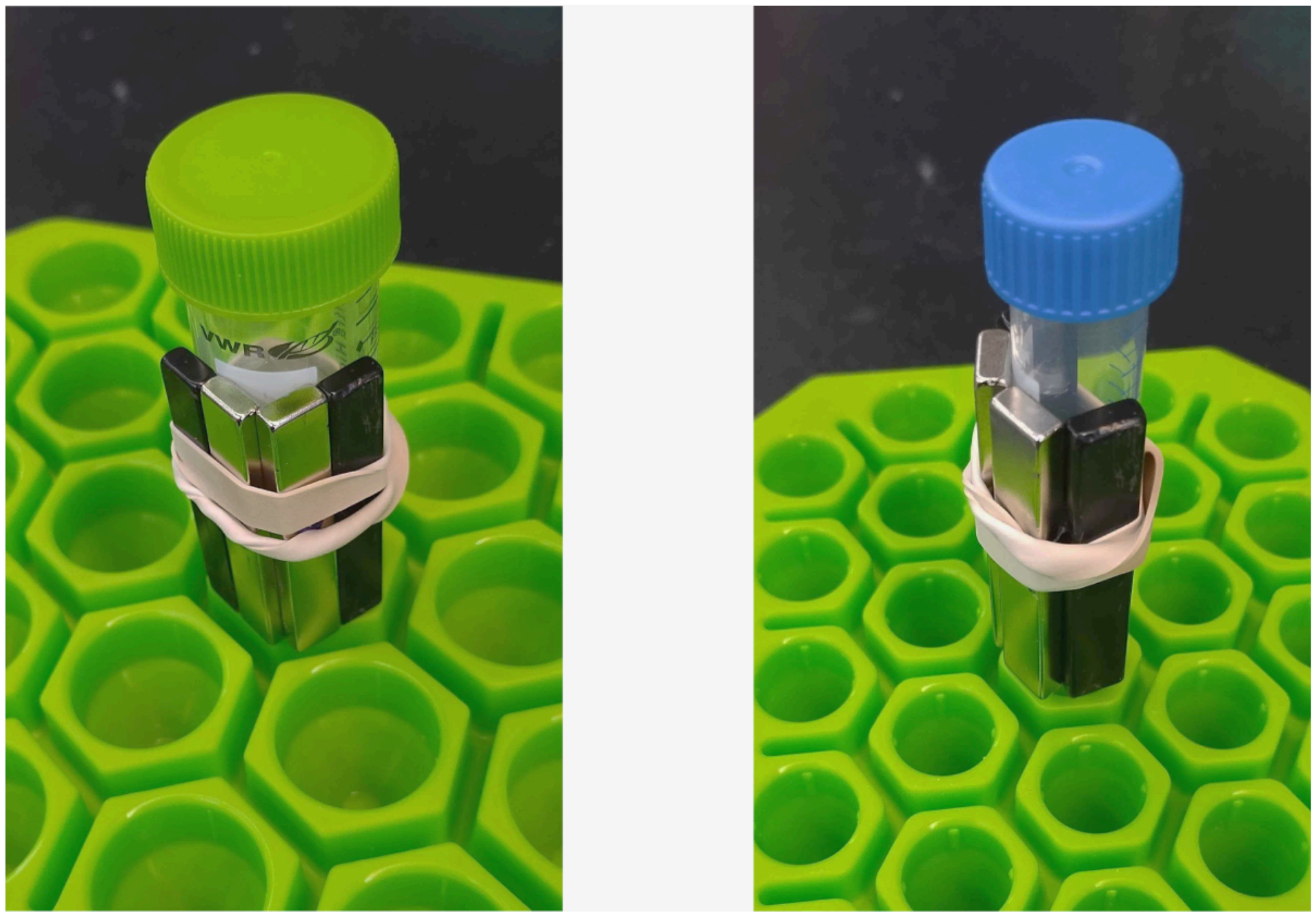

Figure S11: Setup for magnetic separation. Four neodymium magnets of dimensions 60 mm x 10 mm x 5 mm (LOVIMAG # B0CBTSFFSB) are attached to 50 ml (left) or 15 ml (right) falcon tubes using an elastic band.

Table 1: plasmids

Plasmid ID	Insert	Figure	Description	Notes
**pSA52**	AtUBQ10pro::AtRanGAP_WPP-GFP-BLRP	Main Fig. 1B	Constitutive expression of the Arabidopsis RANGAP WPP domain fused to GFP and BRLP	Does not localize to the nuclear envelope
**pUHmSb**	AtUBQ10pro::H2B-mScarlet	Main Fig. 1B	Constitutive expression of Arabidopsis Histone 2B fused to mScarlet	Nuclear marker for microscopy
**pSA7**	AtUBQ10pro:: NbRanGAP_WPP-GFP-BLRP	Main Fig. 1B	Constitutive expression of the N. Benthamiana RANGAP WPP domain fused to GFP and BRLP	Does not localize to the nuclear envelope
**pSA6**	AtUBQ10pro::BLRP-GFP-NbWIP1_KASH	Main Fig. 1B	Constitutive expression of BLRP fused to GFP and the KASH domain of N. Benthamiana WIP1	Does not localize to the nuclear envelope
**pSA266**	6xGal4UAS-min35Spro::H2B-mScarlet-T2A-BLRP-GFP-AtSINE4-C	Extended Data Fig. 1A	Constitutive expression of BLRP fused to GFP and the C terminal domain of Arabidopsis SINE4	Partially localizes to the nuclear envelope
**pSA267**	6xGal4UAS-min35Spro::H2B-mScarlet-T2A-BLRP-GFP-AtTIK-C	Extended Data Fig. 1A	Constitutive expression of BLRP fused to GFP and the C terminal domain of Arabidopsis TIK	Localizes to the nuclear envelope
**pSA268**	6xGal4UAS-min35Spro::H2B-mScarlet-T2A-BLRP-GFP-SlSINE1-C	Extended Data Fig. 1A	Constitutive expression of BLRP fused to GFP and the C terminal domain of tomato SINE1	Partially localizes to the nuclear envelope
**pSA270**	6xGal4UAS-min35Spro::H2B-mScarlet-T2A-BLRP-GFP-SlWIT1-C	Extended Data Fig. 1A	Constitutive expression of BLRP fused to GFP and the C terminal domain of tomato WIT1	Localizes to the nuclear envelope
**pSA272**	6xGal4UAS-min35Spro::H2B-mScarlet-T2A-BLRP-GFP-SlALADIN-C	Extended Data Fig. 1A	Constitutive expression of BLRP fused to GFP and the C terminal domain of tomato ALADIN	Does not localize to the nuclear envelope
**pSA91**	AtUBQ10pro::H2B-mScarlet-T2A-BLRP-GFP-SlWIP1-C	Extended Data Fig. 1B	Constitutive expression of Arabidopsis Histone 2B-mScarlet linked by aT2A peptide to BLRP fused to GFP and the C terminal domain of tomato WIP1	Localizes to the nuclear envelope
**pSA45**	AtUBQ10pro::BLRP-GFP-SlWIP1-C	Main Fig. 1B	Constitutive expression of BLRP fused to GFP and the C terminal domain of tomato WIP1	Localizes to the nuclear envelope
**pSA247**	6xGal4UAS-min35Spro::H2B-mScarlet-T2A-GFP-SlWIP1-C	Extended Data Fig. 1C	Removal of BLRP from reporter	
**pSA246**	6xGal4UAS-min35Spro::H2B-mScarlet-T2A-GFP-SlWIP1-C	Extended Data Fig. 1D	Removal of SlWIP1-C from reporter	
**pSA752**	6xGal4UAS-min35Spro::BLRP-GFP-SlWIP2-C	Extended Data Fig. 1E	BLRP fused to GFP and the C terminal domain of tomato WIP2 driven by Gal4 fusions	GFP accumulates in response to Gal4 activators
**pSA753**	6xGal4UAS-min35Spro::BLRP-GFP-SlWIP1-C	Extended Data Fig. 1E	BLRP fused to GFP and the C terminal domain of tomato WIP1 driven by Gal4 fusions	
**pSA271**	6xGal4UAS-min35Spro::H2B-mScarlet-T2A-BLRP-GFP-SlWIP2-C	Main Fig. 1E	Reporter driven by Gal4 fusions	GFP accumulates in response to Gal4 activators
**pSA225**	6xGal4UAS-min35Spro::H2B-mScarlet-T2A-BLRP-GFP-SlWIP1-C	Main Fig. 1D	Reporter driven by Gal4 fusions	GFP does not accumulate in response to Gal4 activators
**pNH0178**	MASpro::Gal4DBD-AT1G65910	Main Fig. 1G	From Hummel et al. 2023. Gal4 DNA binding domain	
**pNH0274**	MASpro::Gal4DBD-AT1G72010	Main Fig. 1G	From Hummel et al. 2023. Gal4 DNA binding domain	
**pNH0391**	MASpro::Gal4DBD-AT4G25490	Main Fig. 1G	From Hummel et al. 2023. Gal4 DNA binding domain	
**pNH0338**	MASpro::Gal4DBD-AT5G18270	Main Fig. 1G	From Hummel et al. 2023. Gal4 DNA binding domain	
**pNH0400**	MASpro::Gal4DBD-AT5G51990	Main Fig. 1G	From Hummel et al. 2023. Gal4 DNA binding domain	
**p79971**	MASpro::Gal4DBD-VP16	Main Fig. 1G	From Hummel et al. 2023. Gal4 DNA binding domain fused to effector domain	
**p79972**	MASpro::Gal4DBD-VPR	Main Fig. 1G	From Hummel et al. 2023. Gal4 DNA binding domain fused to effector domain	
**p79973**	MASpro::Gal4DBD	Main Fig. 1G	From Hummel et al. 2023. Gal4 DNA binding domain alone	
**pSA75**	AtUBQ10pro::BirA	SI Fig. 1	Constitutive expression of E. Coli BirA codon optimized for N.benthamiana	
**pSA676v2**	6xGal4UAS-min35Spro::mCherry-T2A-BLRP-GFP-SlWIP2-C_AtUBQ10pro::BirA_MASpro::Gal4DBD-dropout	Main Fig. 2C	ENTRAP-seq backbone for cloning tiles as Gal4DBD fusions.	
**pSA676v2s**	6xGal4UAS-min35Spro::mCherry-T2A-BLRP-GFP-SlWIP2-C_AtUBQ10pro::BirA_MASpro::Gal4DBD	Main Fig. 2C	ENTRAP backbone containing the Gal4 DBD not fused to any tiles	
**pSA639**	MASpro::tagBFP2	Main Fig. 2C		
**pSA122**	6xLacO-min35Spro::GFP	Main Fig. 4E	6xLacO GFP reporter for dCas9 recruitment assays	
**pSA401**	AtU6pro::LacOsgRNA1	Main Fig. 4E	sgRNA targeting the LacO sequence driven by the Arabidopsis U6 promoter	
**pAD161**	35Spro::dCas9-VP16	Main Fig. 4E	dCas9 fused to VP16 driven by the 35S promoter	
**pAD162**	35Spro::dCas9-VPR	Main Fig. 4E	dCas9 fused to VPR driven by the 35S promoter	
**pSA742**	35Spro::dCas9-dropout	Main Fig. 4E	dCas9 backbone for cloning tiles	
**pSA14**	AtUBQ10pro::mCherry	Main Fig. 6	For normalization of FTpro::GFP activity	
**pCambia1300**	Empty vector	Main Fig. 6	Empty vector used to replace the CONSTANS expression vector for the 'no CONSTANS' controls	
**pSA615C6**	AtUBQ10pro::tagBFP2-CO	Main Fig. 6	Constitutive expression of BFP fused to wt CONSTANS cds	
**pSA637v2H2**	-1.9Kbp FTpro-GFP	Main Fig. 6	GFP reporter driven by the FLOWERING LOCUS T promoter	

Supplementary Table 2: primers

Primer name	Sequence	Index
**ILidx1-tileSeqF**	AATGATACGGCGACCACCGAGATCTACACgattgctcTCGTCGGCAGCGTCAGATGTGTATAAGAGACAGAAGGTTGCTGGTGGATCTGG	GATTGCTC
**ILidx1-tileSeqR**	CAAGCAGAAGACGGCATACGAGATatagtcggGTCTCGTGGGCTCGGAGATGTGTATAAGAGACAGGAAACTTTATTGCCAAATGTTTGAACG	ATAGTCGG
**ILidx2-tileSeqF**	AATGATACGGCGACCACCGAGATCTACACaccacgatTCGTCGGCAGCGTCAGATGTGTATAAGAGACAGAAGGTTGCTGGTGGATCTGG	ACCACGAT
**ILidx2-tileSeqR**	CAAGCAGAAGACGGCATACGAGATcggtaatcGTCTCGTGGGCTCGGAGATGTGTATAAGAGACAGGAAACTTTATTGCCAAATGTTTGAACG	CGGTAATC
**ILidx3-tileSeqF**	AATGATACGGCGACCACCGAGATCTACACgttgttcgTCGTCGGCAGCGTCAGATGTGTATAAGAGACAGAAGGTTGCTGGTGGATCTGG	GTTGTTCG
**ILidx3-tileSeqR**	CAAGCAGAAGACGGCATACGAGATaaccgtgtGTCTCGTGGGCTCGGAGATGTGTATAAGAGACAGGAAACTTTATTGCCAAATGTTTGAACG	AACCGTGT
**ILidx4-tileSeqF**	AATGATACGGCGACCACCGAGATCTACACcgagactaTCGTCGGCAGCGTCAGATGTGTATAAGAGACAGAAGGTTGCTGGTGGATCTGG	CGAGACTA
**ILidx4-tileSeqR**	CAAGCAGAAGACGGCATACGAGATtcggattcGTCTCGTGGGCTCGGAGATGTGTATAAGAGACAGGAAACTTTATTGCCAAATGTTTGAACG	TCGGATTC
**ILidx5-tileSeqF**	AATGATACGGCGACCACCGAGATCTACACgtgccataTCGTCGGCAGCGTCAGATGTGTATAAGAGACAGAAGGTTGCTGGTGGATCTGG	GTGCCATA
**ILidx5-tileSeqR**	CAAGCAGAAGACGGCATACGAGATtcagacacGTCTCGTGGGCTCGGAGATGTGTATAAGAGACAGGAAACTTTATTGCCAAATGTTTGAACG	TCAGACAC
**ILidx6-tileSeqF**	AATGATACGGCGACCACCGAGATCTACACaactgagcTCGTCGGCAGCGTCAGATGTGTATAAGAGACAGAAGGTTGCTGGTGGATCTGG	AACTGAGC
**ILidx6-tileSeqR**	CAAGCAGAAGACGGCATACGAGATaacacgctGTCTCGTGGGCTCGGAGATGTGTATAAGAGACAGGAAACTTTATTGCCAAATGTTTGAACG	AACACGCT
**ILidx7-tileSeqF**	AATGATACGGCGACCACCGAGATCTACACTACTCATATCGTCGGCAGCGTCAGATGTGTATAAGAGACAGAAGGTTGCTGGTGGATCTGG	TACTCATA
**ILidx7-tileSeqR**	CAAGCAGAAGACGGCATACGAGATTTGGACTTGTCTCGTGGGCTCGGAGATGTGTATAAGAGACAGGAAACTTTATTGCCAAATGTTTGAACG	TTGGACTT
**ILidx8-tileSeqF**	AATGATACGGCGACCACCGAGATCTACACACGCACCTTCGTCGGCAGCGTCAGATGTGTATAAGAGACAGAAGGTTGCTGGTGGATCTGG	ACGCACCT
**ILidx8-tileSeqR**	CAAGCAGAAGACGGCATACGAGATCAGTGGATGTCTCGTGGGCTCGGAGATGTGTATAAGAGACAGGAAACTTTATTGCCAAATGTTTGAACG	CAGTGGAT
**ILidx10-tileSeqF**	AATGATACGGCGACCACCGAGATCTACACCTGATCGTTCGTCGGCAGCGTCAGATGTGTATAAGAGACAGAAGGTTGCTGGTGGATCTGG	CTGATCGT
**ILidx10-tileSeqR**	CAAGCAGAAGACGGCATACGAGATGCGCATATGTCTCGTGGGCTCGGAGATGTGTATAAGAGACAGGAAACTTTATTGCCAAATGTTTGAACG	GCGCATAT
**ILidx11-tileSeqF**	AATGATACGGCGACCACCGAGATCTACACCGGCTAATTCGTCGGCAGCGTCAGATGTGTATAAGAGACAGAAGGTTGCTGGTGGATCTGG	CGGCTAAT
**ILidx11-tileSeqR**	CAAGCAGAAGACGGCATACGAGATCTCGTTCTGTCTCGTGGGCTCGGAGATGTGTATAAGAGACAGGAAACTTTATTGCCAAATGTTTGAACG	CTCGTTCT
**ILidx12-tileSeqF**	AATGATACGGCGACCACCGAGATCTACACGACGATCTTCGTCGGCAGCGTCAGATGTGTATAAGAGACAGAAGGTTGCTGGTGGATCTGG	GACGATCT
**ILidx12-tileSeqR**	CAAGCAGAAGACGGCATACGAGATTCGTGCATGTCTCGTGGGCTCGGAGATGTGTATAAGAGACAGGAAACTTTATTGCCAAATGTTTGAACG	TCGTGCAT
**ILidx13-tileSeqF**	AATGATACGGCGACCACCGAGATCTACACCCTTGATCTCGTCGGCAGCGTCAGATGTGTATAAGAGACAGAAGGTTGCTGGTGGATCTGG	CCTTGATC
**ILidx13-tileSeqR**	CAAGCAGAAGACGGCATACGAGATGATGGAGTGTCTCGTGGGCTCGGAGATGTGTATAAGAGACAGGAAACTTTATTGCCAAATGTTTGAACG	GATGGAGT
**tileLib-F**	ACGTCAGTGTGGTCTCTAGGT	N/a
**tileLib-R**	TAAGCGCTTGGTCTCTAAGCTTA	N/a
