# Supplementary material for: Multiplexed profiling of transcriptional regulators in plant cells
Source: Nat Biotechnol. 2025 Nov 25;44(11):1920–33. doi: 10.1038/s41587-025-02880-w (PMC13751309; doi:10.1038/s41587-025-02880-w)
Supplement: Supplementary file 2 [file 41587_2025_2880_MOESM2_ESM.pdf]

nature portfolio

Corresponding author(s): Patrick M. Shih.

Last updated by author(s): Sep 20, 2025

Reporting Summary

Nature Portfolio wishes to improve the reproducibility of the work that we publish. This form provides structure for consistency and transparency in reporting. For further information on Nature Portfolio policies, see our Editorial Policies and the Editorial Policy Checklist.

Statistics

For all statistical analyses, confirm that the following items are present in the figure legend, table legend, main text, or Methods section.

n/a	Confirmed	
☐	☒	The exact sample size (*n*) for each experimental group/condition, given as a discrete number and unit of measurement
☐	☒	A statement on whether measurements were taken from distinct samples or whether the same sample was measured repeatedly
☐	☒	The statistical test(s) used AND whether they are one- or two-sided<br>*Only common tests should be described solely by name; describe more complex techniques in the Methods section.*
☒	☐	A description of all covariates tested
☐	☒	A description of any assumptions or corrections, such as tests of normality and adjustment for multiple comparisons
☐	☒	A full description of the statistical parameters including central tendency (e.g. means) or other basic estimates (e.g. regression coefficient) AND variation (e.g. standard deviation) or associated estimates of uncertainty (e.g. confidence intervals)
☐	☒	For null hypothesis testing, the test statistic (e.g. *F*, *t*, *r*) with confidence intervals, effect sizes, degrees of freedom and *P* value noted<br>*Give P values as exact values whenever suitable.*
☒	☐	For Bayesian analysis, information on the choice of priors and Markov chain Monte Carlo settings
☒	☐	For hierarchical and complex designs, identification of the appropriate level for tests and full reporting of outcomes
☐	☒	Estimates of effect sizes (e.g. Cohen's *d*, Pearson's *r*), indicating how they were calculated

Our web collection on statistics for biologists contains articles on many of the points above.

Software and code

Policy information about availability of computer code

Data collection	Standard proprietary Illumina software was used by the sequencing provider to obtain the reads for each library. Fluorescence measurements were performed using the BioTek Gen5 software.
Data analysis	Read mapping was performed using 2FAST2Q version 2.6.0. The rest of data analysis was performed using custom-made Python scripts. All code and models related to this study are publicly available on GitHub (github.com/shih-lab/magnets).

For manuscripts utilizing custom algorithms or software that are central to the research but not yet described in published literature, software must be made available to editors and reviewers. We strongly encourage code deposition in a community repository (e.g. GitHub). See the Nature Portfolio guidelines for submitting code & software for further information.

Data

Policy information about availability of data

All manuscripts must include a data availability statement. This statement should provide the following information, where applicable:

- Accession codes, unique identifiers, or web links for publicly available datasets
- A description of any restrictions on data availability
- For clinical datasets or third party data, please ensure that the statement adheres to our policy

Raw sequencing reads are publicly available through the NCBI SRA Database under BioProject accession PRJNA1254296. Raw and processed fluorescence data is provided as supplementary material. Read enrichment ratios for each experiment are provided as supplementary material.

Research involving human participants, their data, or biological material

Policy information about studies with human participants or human data. See also policy information about sex, gender (identity/presentation), and sexual orientation and race, ethnicity and racism.

Reporting on sex and gender	not applicable
Reporting on race, ethnicity, or other socially relevant groupings	not applicable
Population characteristics	not applicable
Recruitment	not applicable
Ethics oversight	not applicable

Note that full information on the approval of the study protocol must also be provided in the manuscript.

Field-specific reporting

Please select the one below that is the best fit for your research. If you are not sure, read the appropriate sections before making your selection.

☒ Life sciences ☐ Behavioural & social sciences ☐ Ecological, evolutionary & environmental sciences

For a reference copy of the document with all sections, see nature.com/documents/nr-reporting-summary-flat.pdf

Life sciences study design

All studies must disclose on these points even when the disclosure is negative.

Sample size	The sample size for fluorescence measurements was N = 24 or 32 biological replicates. A biological replicate is defined as a tissue area from an infiltrated leaf. For N = 24, 3 plants, 2 leaves per plant, 4 areas per leaf. For N = 32, 4 plants, 2 leaves per plant, 4 areas per leaf. This sample size was chosen so as to match or exceed the number of biological replicates typically used in Agroinfiltration experiments in N. benthamiana, which are usually in the order of 9-16 biological replicates (Hummel et al. 2024, Belcher et al. 2020, Brophy et al. 2022). This number of replicates ensures robust detection of relevant small biological differences. Because of time constraints we used 24 replicates for very large experiments involving many infiltrations and 32 replicates otherwise. A recent power analysis of this experimental setup demonstrated that N=24 is sufficient to detect statistically significant differences of 10-20% between means (Tang et al. BioRxiv 2025).<br>For library sequencing experiments we performed 2 replicates. This number was chosen because it is the minimum number of replicates necessary to assess reproducibility. Because of the effort and cost of these sequencing experiments we did not perform more replicates, nonetheless, all past comparable studies (see citations in the main text) performed 1 or 2 replicates at most.
Data exclusions	No data were excluded in any of the main figures except for Fig. 3C. Data exclusion was necessary for this figure because it shows the effect of excluding underrepresented tiles on assay reproducibility. Also, our comparison of the dynamic range of ENTRAP-seq versus Ludwig et al.shown in Supplementary Figure S10 excluded outliers (<0.01 and >0.99 percentiles).
Replication	All attempts at replication were successful. Fluorescence measurements were successfully replicable, as demonstrated by the statistical tests applied in each case. The replication of sequencing experiments is analyzed in depth in the manuscript, particularly Fig. 3C which shows that two biological replicates of these experiments are reproducible. TFigure 3D shows that tile activity as measured by enrichment is reproducible across different libreries. The reproducibility of tile enrichment is also described in detail in Extended Data Fig. 4 and the supplementary text section "Resolution and reproducibility of ENTRAP-seq measurements."
Randomization	In fluorescence assays, plants, leaves and areas within the leaf were preallocated randomly to different treatments (i.e, infiltrations). Similarly, Agrobacterium cultures carrying libraries were preallocated randomly to different plants. To account for possible unknown covariates in fluorescence reporter assays, a normalization Agrobacterium strain was coinfiltrated and used for signal normalization.
Blinding	We blinded ourselves by analyzing all fluorescence and sequencing data was in bulk, simultaneously, using a common shared computational pipeline, without prior knowledge of sample identity.

Reporting for specific materials, systems and methods

We require information from authors about some types of materials, experimental systems and methods used in many studies. Here, indicate whether each material, system or method listed is relevant to your study. If you are not sure if a list item applies to your research, read the appropriate section before selecting a response.

Materials & experimental systems

n/a	Involved in the study
☒	☐ Antibodies
☒	☐ Eukaryotic cell lines
☒	☐ Palaeontology and archaeology
☒	☐ Animals and other organisms
☒	☐ Clinical data
☒	☐ Dual use research of concern
☒	☐ Plants

Methods

n/a	Involved in the study
☒	☐ ChIP-seq
☒	☐ Flow cytometry
☒	☐ MRI-based neuroimaging

Plants

Seed stocks	N. benthamiana wild type was obtained from the JBEI registry and is publicly available.
Novel plant genotypes	No novel plant genotypes were generated.
Authentication	Not applicable (no novel plant genotypes were generated.)
